# Crystal structure of 1-[(4-methyl­phen­yl)sulfon­yl]-2-phenyl­azetidine

**DOI:** 10.1107/S2056989026010315

**Published:** 2026-10-11

**Authors:** Berthy J. Aroche, Katherine Barraza Hernandez, Zoe Y. Marr, S. Chantal E. Stieber, Taylor A. Thane

**Affiliations:** a Department of Chemistry & Biochemistry California State Polytechnic University, Pomona 3801 W Temple Ave Pomona CA 91768 USA

**Keywords:** crystal structure, azetidine, 2-phenyl-*N*-tosyl­azetidine, supramolecular structure

## Abstract

The crystal structure of 1-[(4-methyl­phen­yl)sulfon­yl]-2-phenyl­azetidine, C_16_H_17_NO_2_S, commonly referred to as 2-phenyl-*N*-tosyl­azetidine, is reported. There are three close con­tacts stabilizing the supra­molecular structure. Two of the close con­tacts are between one of the O atoms on the sulfonamide moiety and two neighboring C—H bonds from the tolyl ring. The third close con­tact is between the H—C bond of the azetidine and a C atom on the phenyl ring. Distances between the tolyl rings are greater than the standard distance of 5 Å for π-stacking.

## Abstract 2

In the crystal structure of 2-phenyl-*N*-tosyl­azetidine, there are three close con­tacts stabilizing the supra­molecular structure.

## 1. Chemical con­text

Azetidines, highly strained four-membered nitro­gen-con­taining heterocycles, serve as unique linchpin motifs due to their prevalence in biologically relevant com­pounds and their propensity for ring-opening reaction pathways (Alberto *et al.*, 2008 ▸). As such, the synthesis of new azetidine motifs is necessary for the development of novel pharmaceutical and agrochemical com­pounds and has garnered a resurging inter­est by synthetic and medicinal chemists alike (Bauer *et al.*, 2021 ▸). This resurgence is due to unique physiochemical and pharmokinetic properties which make them attractive targets for drug development (Cheekatla, 2026 ▸). As such, azetidines are used strategically in drug development as bioisteres for their five- and six-membered ring counterparts, such as piperidines and pyrrolidines (Feskov *et al.*, 2019 ▸; Meanwell & Loiseleur, 2022 ▸; Kirichock *et al.*, 2024 ▸). This renewed inter­est in the azetidine moiety is evident by the increased number of FDA approved drugs that con­tain an azetidine moiety over the last ten years (Gelato *et al.*, 2026 ▸). Examples of small-mol­ecule drugs that con­tain an azetidine moiety include tebanicline, a non-opioid analgesic, ximelagatran, an anti­coagulant, and delafloxacin, an anti­biotic (Parmar *et al.*, 2021 ▸; Mughal & Szostak, 2021 ▸).

Efficient synthetic approaches that install the azetidine motif are required to meet the demand of medicinal chemists. The most common strategies include intra­molecular nucleophilic substitution reactions and UV- or blue-light-mediated aza-Paternò–Büchi reactions (Brandi *et al.*, 2008 ▸; Richardson *et al.*, 2020 ▸). Another common approach to accessing azetidine motifs includes functionalizing 3-azetidinones which allows for a variety of functionalization reactions, such as a Wittig addition or Grignard addition at the ketone (Dejaegher *et al.*, 2002 ▸). Alternatively, 2-azetidinones, which are building blocks for β-lactams, can be reduced to yield the desired azetidine (Alcaide *et al.*, 2013 ▸). Cumulatively, these synthetic methods rely on pre-functionalized starting materials and classic synthetic approaches.

Herein, we report the crystal structure of 1-[(4-methyl­phen­yl)sulfon­yl]-2-phenyl­azetidine (2-phenyl-*N*-tosyl­az­e­ti­dine). 2-Phenyl-*N*-tosyl­azetidine was synthesized *via* a two-step synthesis from β-phenyl­alanine. β-Phenyl­alanine was sub­jected to a lithium aluminium hydride reduction in quantative yield and was carried forward without further purification into an intra­molecular substitution reaction which yielded the desired 2-phenyl-*N*-tosyl­azetidine.
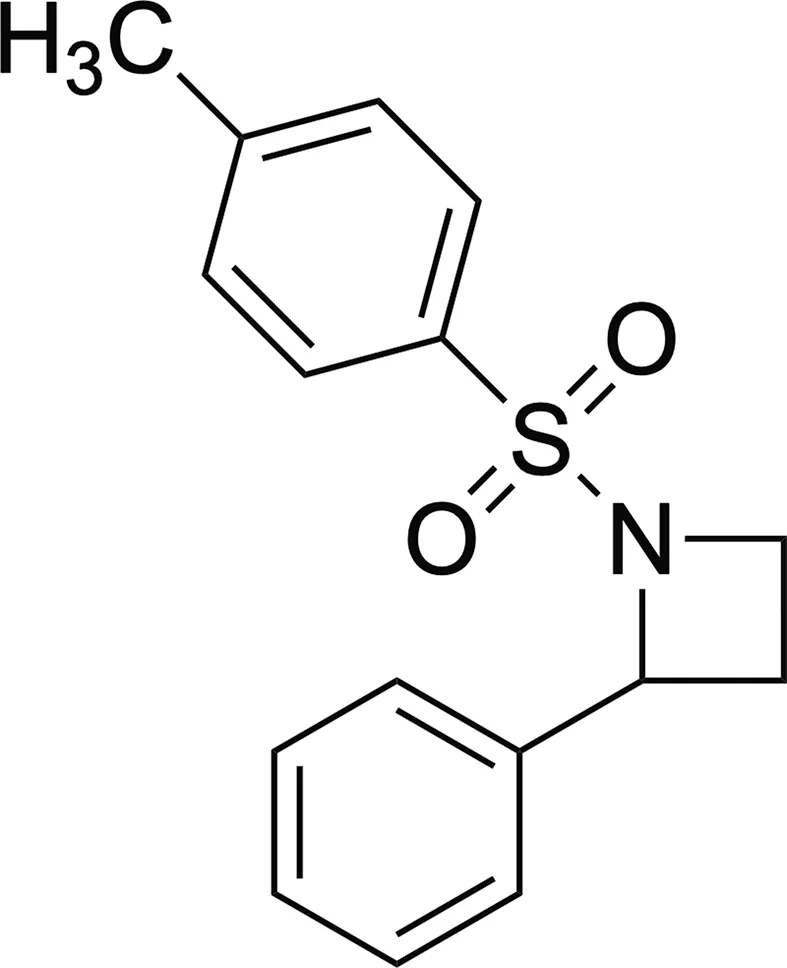


## 2. Structural commentary

**Figure 1:**
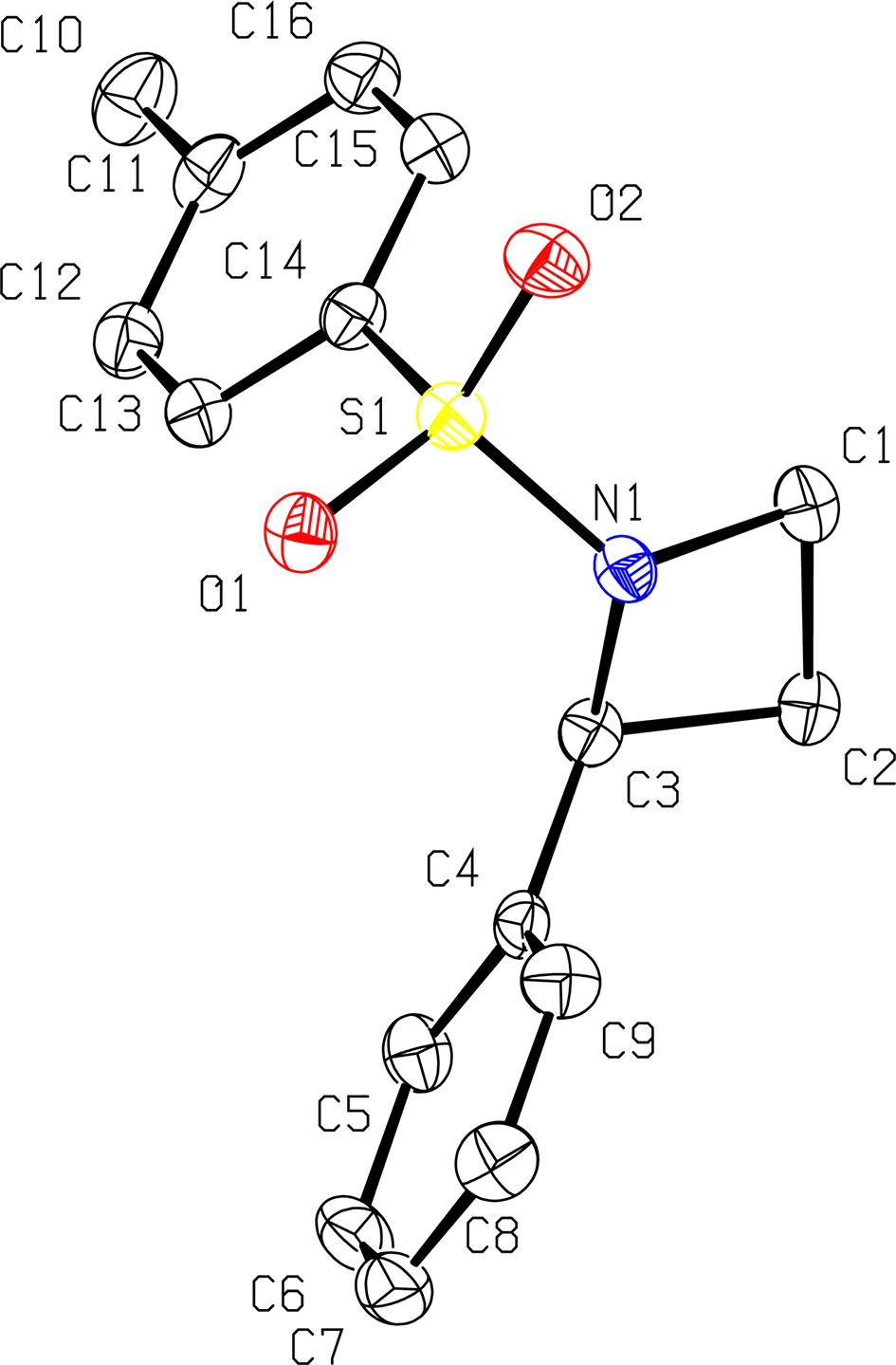
View of one 2-phenyl-*N*-tosyl­azetidine mol­ecule drawn with 50% probability displacement ellipsoids.

The structural features for 2-phenyl-*N*-tosyl­azetidine as shown in Fig. 1 ▸ are reported below. The bond lengths of the azetidine ring are N1—C1 = 1.500 (2) Å, N1—C3 = 1.499 (2) Å, C1—C2 = 1.539 (3) Å, and C2—C3 = 1.552 (3) Å. The bond distances within the azetidine ring are consistent with literature reports (Taggi *et al.*, 2000 ▸). A related com­pound, 2-(4-bromo­phen­yl)-1-[(4-methyl­phen­yl)sulfon­yl]azetidine, reports C—N bond lengths of 1.567 and 1.488 Å, respectivly, and C—C bond lengths of 1.648 and 1.474 Å, which are consistent with the title com­pound (Malik & Pandey, 2022 ▸). The bond lengths of the sulfonamide are N1—S1 = 1.6319 (16) Å, S1—O1 = 1.4340 (14) Å, S1—O2 = 1.4361 (14) Å, and S1—C14 = 1.761 (2) Å. The bond lengths of the S atom on the sulfonamide moiety are consistent with literature values from prior reports (Syed *et al.*, 2011 ▸). Similar bond lengths for the sulfonamide group of the related com­pound are reported as follows: S—N = 1.611 Å, S—C = 1.749 Å, S—O1 = 1.462 Å, and S—O2 = 1.452 Å (Malik & Pandey, 2022 ▸).

The bond angles within the azetidine ring are C3—N1—C1 = 91.48 (14)°, N1—C1—C2 = 87.70 (14)°, C1—C2—C3 = 88.04 (14)°, and N1—C3—C2 = 87.29 (13)°, which are consistent with prior reports for *N*-tosyl­azetidines (Taggi *et al.*, 2000 ▸). The related com­pound 2-(4-bromo­phen­yl)-1-[(4-methyl­phen­yl)sulfon­yl]azetidine reports similar bond angles within the azetidine moiety, with a C—N—C bond angle of 93.21° (Malik & Pandey, 2022 ▸). The bond angles around the S atom of the sulfonamide moiety are O1—S1—O2 = 120.64 (9)°, O1—S1—N1 = 106.31 (8)°, O1—S1—C14 = 107.76 (9)°, O2—S1—N1 = 105.86 (8)°, O2—S1—C14 = 108.26 (9)°, and N1—S1—C14 = 107.25 (9)°, which are also consistent with prior reports (Syed *et al.*, 2011 ▸). Bond angles around the sulfonamide moiety of the related com­pound include an O—S—O angle of 121.94°, O—S—N angles of 98.82 and 112.22°, and a C—S—N angle of 107.09° which are consistent with the title com­pound (Malik & Pandey, 2022 ▸)

## 3. Supra­molecular features

**Figure 2:**
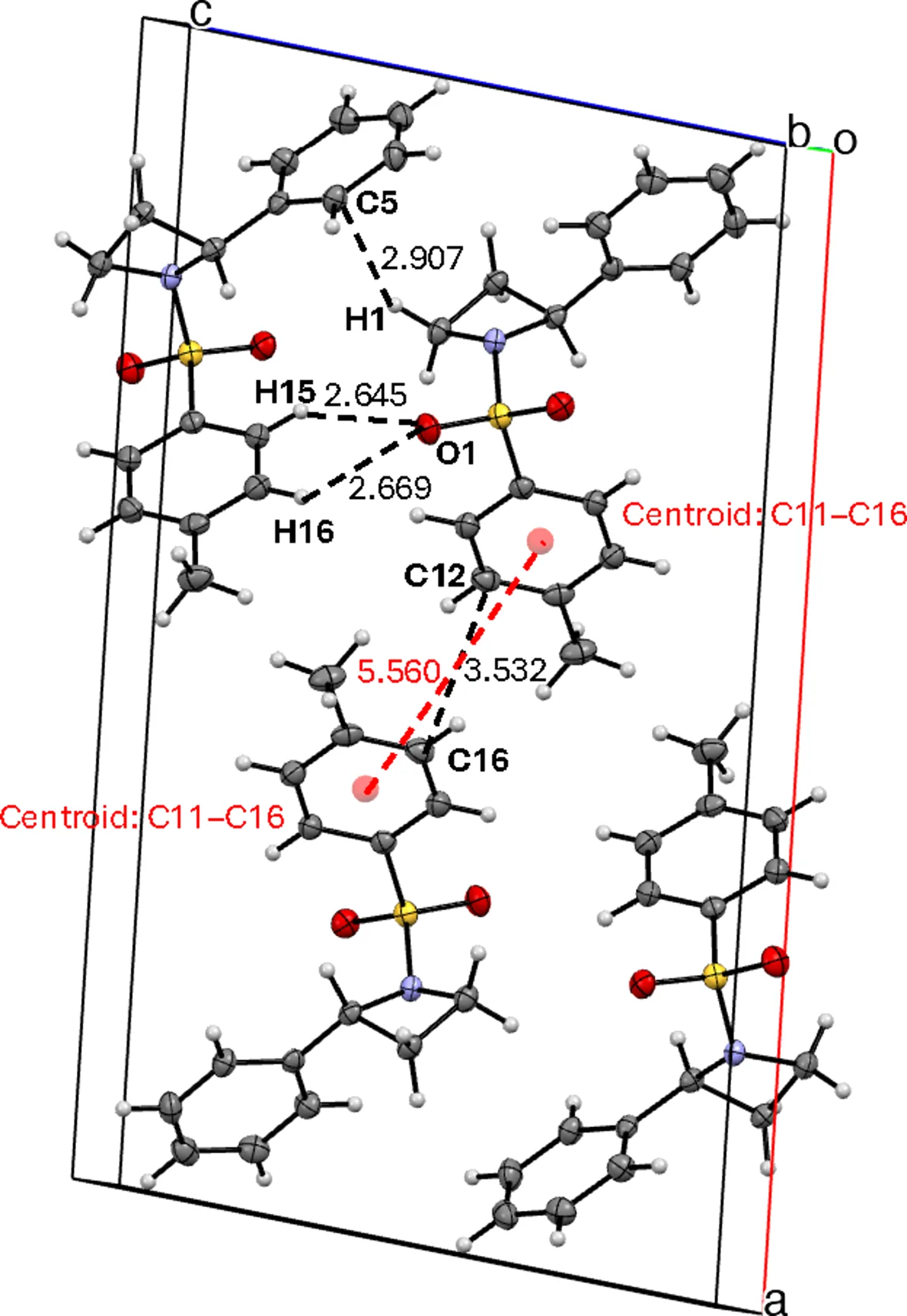
The unit cell with 50% probability displacement ellipsoids shows four 2-phenyl-*N*-tosyl­azetidine mol­ecules. Mol­ecular distances and close con­tacts (Å) are repre­sent­ed in black. Distances between centroids (red circles) are calculated.

Four 2-phenyl-*N*-tosyl­azetidine mol­ecules are shown in the unit cell (Fig. 2 ▸). Three H⋯*X* bonds stablize the supra­molecular structure, with the corresponding distances indicating hy­dro­gen-bonding inter­actions. The H15⋯O1 in­ter­action is at a distance of 2.645 Å, the H16⋯O1 inter­action is at a distance of 2.669 Å, and the H1⋯C5 inter­action is at a distance of 2.907 Å. Additionally, there is a relatively close C12⋯C13 distance of 3.532 Å. The distance between the centroid of the C11–C16 aryl ring and another C11–C16 centroid on a neighboring mol­ecule is 5.560 Å, which is outside the range expected for π-stacking (Janiak, 2000 ▸). The centroid algorithm in the program *Mercury* was used to place the centroids resulting in the measured distance (Macrae *et al.*, 2020 ▸).

## 4. Database survey

A database search in the Cambridge Structural Database (WebCSD, Version of 2026) on 15 June 2026 produced no structural results (Groom *et al.*, 2016 ▸). Additionally, a search in *Scifinder* (Scifinder, 2026 ▸) yielded a com­pound match for the racemic com­pound 1-[(4-methyl­phen­yl)sulfon­yl]-2-phenyl­azetidine (code 38455-36-6). Matches for both the *S* and *R* enanti­omers, (2*S*)-1-[(4-methyl­phen­yl)sulfon­yl]-2-phenyl­azeti­dine (code 936011-91-5) and (2*R*)-1-[(4-methyl­phen­yl)sulfon­yl]-2-phenyl­azetidine (code 1079366-88-3), were also found. While both racemic and enanti­opure forms of the structure have been synthesized and characterized, no structural data has been reported. However, a related com­pound, 2-(4-bromo­phen­yl)-1-[(4-methyl­phen­yl)sulfon­yl]azetidine, has been re­por­ted with bond angles and bond lengths similar to those determined for the title azetidine, 2-phenyl-*N*-tosyl­azetidine (deposition No. 762724; Malik & Pandey, 2022 ▸).

## 5. Synthesis and crystallization

**Figure 3:**
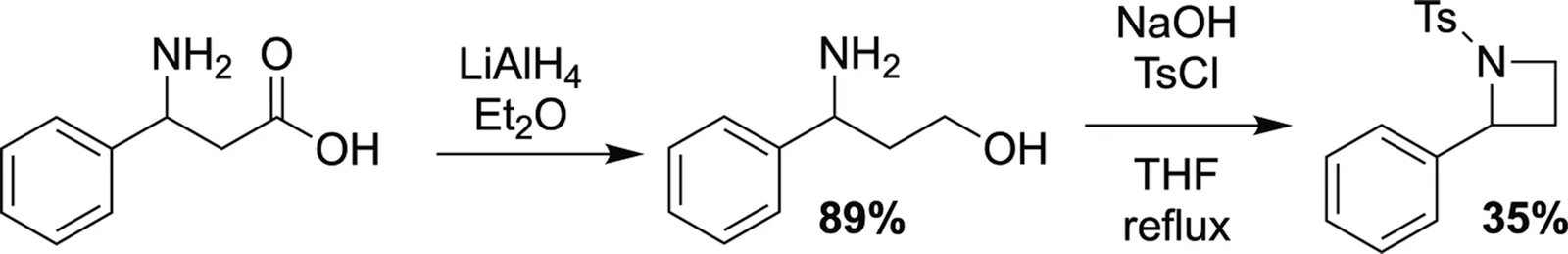
The synthesis of 2-phenyl-*N*-tosyl­azetidine.

2-Phenyl-*N*-tosyl­azetidine was synthesized in two steps (Fig. 3 ▸). The first step was a lithium aluminium hydride reduction of β-phenyl­alanine that was modified from the literature (Sanford *et al.*, 2020 ▸). A flame-dried round-bottomed flask equipped with a stir bar was charged with β-phenyl­alanine (3.31 g, 20.0 mmol, 1.00 equiv.) and tetra­hydro­furan (THF; 100 ml, 0.2 *M* in substrate). The reaction flask was cooled to 0 °C and lithium aluminium hydride (16.5 ml, 66.0 mmol, 3.30 equiv., 4.00 *M* solution in THF) was added dropwise. The reaction flask was warmed to room tem­per­a­ture and stirred overnight. The reaction was worked up according to the Fieser workup, where *X* ml is equal to *X* grams of LiAlH_4_ used (Chandra & Zebrowski, 2024 ▸). For example, in this reaction, *X* grams of lithium aluminium hydride equals 2.5 g (66 mmol), so 2.5 ml of water (*X* ml), 2.5 ml of 15% aqueous NaOH solution (*X* ml), and 7.5 ml of water (3*X* ml) were added respectively as described below. The reaction was diluted with neat Et_2_O and cooled to 0 °C. Water (2.5 ml, *X* ml) was added slowly, followed by the addition of 15% aqueous NaOH (2.5 ml, *X* ml) and then by a second slow addition of water (7.5 ml, 3*X* ml). The reaction was allowed to warm to room tem­per­a­ture and left to stir for 15 min. Mg_2_SO_4_ was then added to the reaction mixture and left to stir for an additional 15 min. The precipitates were removed by vacuum filtration and the filtrate was concentrated *in vacuo*. The white solid, γ-amino­benzene­propanol, was carried forward without further purification (2.68 g, 17.8 mmol, 89% yield). ^1^H NMR analysis is consistent with literature reports (Torre *et al.*, 2006 ▸).

The second step, an intra­molecular substitution of γ-amino­benzene­propanol, was also adapted from prior reports (Wang *et al.*, 2026 ▸). To a flamed-dried round-bottomed flask equipped with a stir bar, NaOH (0.48 g, 12 mmol, 3.0 equiv.) and THF (6 ml, 2 *M* in NaOH) were added. In a separate flame-dried round-bottomed flask, a solution of γ-amino­benzene­propanol (0.61 g, 4.0 mmol, 1.0 equiv.) was dissolved in THF (10 ml, 0.4 *M* in γ-amino­benzene­propanol) and added to the reaction mixture dropwise at room tem­per­a­ture. Next, toluene­sulfonic acid (0.84 g, 4.4 mmol, 1.1 equiv.) was added to the reaction mixture in portions. The reaction mixture was then heated to reflux and allowed to stir for 3 h. After com­pletion, the reaction mixture was diluted with cold deionized water (30 ml) and extracted with EtOAc (×3). The combined organic layers were washed with brine, dried over Na_2_SO_4_, and concentrated *in vacuo*. The resulting oil was purified by flash column chromatography (0–25% EtOAc/hexa­nes) to afford a white solid (0.398 g, 1.39 mmol, 35% yield). ^1^H NMR analysis is consistent with literature reports (Prasad *et al.*, 2004 ▸). The melting-point range was 103–105 °C. A singular crystal was grown *via* vapor diffusion by dissolving 10 mg of 2-phenyl-*N*-tosyl­azetidine in 0.5 ml of ethyl acetate. Ethyl acetate was used as the solvent and hexa­nes as the anti-solvent.

## 6. Refinement

Crystal data, data collection and structure refinement details are summarized in Table 1 ▸. H atoms that were tertiary CH and secondary CH_2_ were refined using a riding model. The methyl group was refined as an idealized rotating methyl group. Fixed atomic displacement parameters [*U*_iso_(H)] were set at 1.2 times *U*_iso_(C) for all CH and CH_2_ groups, and 1.5 times for all CH_3_ groups.

## Supplementary Material

Crystal structure: contains datablock(s) I, global. DOI: 10.1107/S2056989026010315/meu2006sup1.cif

Structure factors: contains datablock(s) I. DOI: 10.1107/S2056989026010315/meu2006Isup3.hkl

Supporting information file. DOI: 10.1107/S2056989026010315/meu2006Isup3.cml

CCDC reference: 2593072

Additional supporting information:  crystallographic information; 3D view; checkCIF report

## Figures and Tables

**Table 1:** Experimental details

Crystal data	
Chemical formula	C_16_H_17_NO_2_S
*M*_r_	287.36
Crystal system, space group	Monoclinic, *P*2_1_/*c*
Temperature (K)	127
*a*, *b*, *c* (Å)	21.3441 (12), 5.5024 (3), 12.1775 (6)
β (°)	97.856 (2)
*V* (Å^3^)	1416.75 (13)
*Z*	4
Radiation type	Mo *K*α
μ (mm^−1^)	0.23
Crystal size (mm)	0.13 × 0.10 × 0.02

Data collection	
Diffractometer	Bruker APEXII CCD
Absorption correction	Multi-scan (*SADABS*; (Bruker, 2016 ▸)
*T*_min_, *T*_max_	0.667, 0.746
No. of measured, independent and observed [*I* > 2σ(*I*)] reflections	42002, 3588, 2531
*R*_int_	0.114
(sin θ/λ)_max_ (Å^−1^)	0.671

Refinement	
*R*[*F*^2^ > 2σ(*F*^2^)], *wR*(*F*^2^), *S*	0.044, 0.112, 1.05
No. of reflections	3588
No. of parameters	182
H-atom treatment	H-atom parameters constrained
Δρ_max_, Δρ_min_ (e Å^−3^)	0.24, −0.47

Computer programs: *APEX2* (Bruker, 2019 ▸), *SAINT* (Bruker, 2019 ▸), *SHELXT2018* (Sheldrick, 2015*a* ▸), SHEXL2019 (Sheldrick, 2015*b* ▸), and *OLEX2* (Dolomanov *et al.*, 2009 ▸).

## supplementary crystallographic information

### 1-[(4-Methylphenyl)sulfonyl]-2-phenylazetidine Crystal data

**Table:**

C_16_H_17_NO_2_S	*F*(000) = 608
*M**_r_* = 287.36	*D*_x_ = 1.347 Mg m^−^^3^
Monoclinic, *P*2_1_/*c*	Mo *K*α radiation, λ = 0.71073 Å
*a* = 21.3441 (12) Å	Cell parameters from 4338 reflections
*b* = 5.5024 (3) Å	θ = 2.9–26.2°
*c* = 12.1775 (6) Å	µ = 0.23 mm^−^^1^
β = 97.856 (2)°	*T* = 127 K
*V* = 1416.75 (13) Å^3^	Plate, colourless
*Z* = 4	0.13 × 0.10 × 0.02 mm

### 1-[(4-Methylphenyl)sulfonyl]-2-phenylazetidine Data collection

**Table:**

Bruker APEXII CCD diffractometer	2531 reflections with *I* > 2σ(*I*)
φ and ω scans	*R*_int_ = 0.114
Absorption correction: multi-scan (SADABS; (Bruker, 2016)	θ_max_ = 28.5°, θ_min_ = 2.9°
*T*_min_ = 0.667, *T*_max_ = 0.746	*h* = −28→28
42002 measured reflections	*k* = −7→7
3588 independent reflections	*l* = −16→16

### 1-[(4-Methylphenyl)sulfonyl]-2-phenylazetidine Refinement

**Table:**

Refinement on *F*^2^	Primary atom site location: dual
Least-squares matrix: full	Hydrogen site location: inferred from neighbouring sites
*R*[*F*^2^ > 2σ(*F*^2^)] = 0.044	H-atom parameters constrained
*wR*(*F*^2^) = 0.112	*w* = 1/[σ^2^(*F*_o_^2^) + (0.0334*P*)^2^ + 1.1271*P*] where *P* = (*F*_o_^2^ + 2*F*_c_^2^)/3
*S* = 1.05	(Δ/σ)_max_ < 0.001
3588 reflections	Δρ_max_ = 0.24 e Å^−^^3^
182 parameters	Δρ_min_ = −0.47 e Å^−^^3^
0 restraints	

### 1-[(4-Methylphenyl)sulfonyl]-2-phenylazetidine Special details

**Table:**

Geometry. All esds (except the esd in the dihedral angle between two l.s. planes) are estimated using the full covariance matrix. The cell esds are taken into account individually in the estimation of esds in distances, angles and torsion angles; correlations between esds in cell parameters are only used when they are defined by crystal symmetry. An approximate (isotropic) treatment of cell esds is used for estimating esds involving l.s. planes.

### 1-[(4-Methylphenyl)sulfonyl]-2-phenylazetidine Fractional atomic coordinates and isotropic or equivalent isotropic displacement parameters (Å^2^)

**Table:**

	*x*	*y*	*z*	*U*_iso_*/*U*_eq_	
S1	0.27972 (2)	0.88010 (8)	0.42309 (4)	0.02094 (13)	
O1	0.26099 (7)	1.0097 (2)	0.32175 (11)	0.0257 (3)	
O2	0.30263 (7)	1.0082 (3)	0.52331 (11)	0.0289 (3)	
N1	0.21746 (7)	0.7304 (3)	0.44931 (12)	0.0200 (3)	
C1	0.21859 (10)	0.5633 (4)	0.54692 (16)	0.0239 (4)	
H1A	0.203627	0.639064	0.612336	0.029*	
H1B	0.259478	0.479016	0.567666	0.029*	
C2	0.16829 (9)	0.4087 (4)	0.47600 (15)	0.0228 (4)	
H2A	0.124461	0.441139	0.490053	0.027*	
H2B	0.177494	0.232241	0.478166	0.027*	
C3	0.18662 (9)	0.5398 (3)	0.37217 (15)	0.0196 (4)	
H3	0.219346	0.444061	0.339458	0.024*	
C4	0.13608 (9)	0.6240 (3)	0.28295 (15)	0.0195 (4)	
C5	0.12295 (10)	0.4868 (4)	0.18672 (16)	0.0245 (4)	
H5	0.146691	0.343721	0.178011	0.029*	
C6	0.07557 (11)	0.5567 (4)	0.10338 (17)	0.0307 (5)	
H6	0.066834	0.460759	0.038214	0.037*	
C7	0.04093 (10)	0.7656 (4)	0.11469 (17)	0.0297 (5)	
H7	0.008537	0.813451	0.057377	0.036*	
C8	0.05369 (10)	0.9048 (4)	0.20987 (18)	0.0293 (5)	
H8	0.030168	1.048721	0.217865	0.035*	
C9	0.10075 (10)	0.8338 (4)	0.29336 (17)	0.0262 (4)	
H9	0.109089	0.929486	0.358671	0.031*	
C10	0.46824 (11)	0.1036 (4)	0.3375 (2)	0.0353 (5)	
H10A	0.508539	0.127096	0.385503	0.053*	
H10B	0.475353	0.108096	0.259753	0.053*	
H10C	0.450226	−0.054081	0.353719	0.053*	
C11	0.42296 (9)	0.3035 (4)	0.35857 (18)	0.0261 (4)	
C12	0.38082 (10)	0.3990 (4)	0.27229 (17)	0.0258 (4)	
H12	0.381427	0.340038	0.199118	0.031*	
C13	0.33794 (9)	0.5783 (4)	0.29077 (16)	0.0236 (4)	
H13	0.309414	0.641763	0.230925	0.028*	
C14	0.33712 (9)	0.6646 (3)	0.39812 (16)	0.0217 (4)	
C15	0.37905 (9)	0.5731 (4)	0.48549 (16)	0.0251 (4)	
H15	0.378530	0.632727	0.558586	0.030*	
C16	0.42172 (10)	0.3940 (4)	0.46518 (17)	0.0284 (5)	
H16	0.450587	0.331957	0.524898	0.034*	

### 1-[(4-Methylphenyl)sulfonyl]-2-phenylazetidine Atomic displacement parameters (Å^2^)

**Table:**

	*U*^11^	*U*^22^	*U*^33^	*U*^12^	*U*^13^	*U*^23^
S1	0.0236 (2)	0.0173 (2)	0.0216 (2)	−0.0011 (2)	0.00212 (18)	−0.00076 (19)
O1	0.0297 (8)	0.0205 (7)	0.0271 (8)	0.0012 (6)	0.0047 (6)	0.0052 (6)
O2	0.0332 (8)	0.0251 (8)	0.0272 (8)	−0.0047 (6)	−0.0002 (6)	−0.0081 (6)
N1	0.0232 (8)	0.0200 (8)	0.0169 (8)	−0.0003 (7)	0.0029 (6)	−0.0011 (6)
C1	0.0285 (11)	0.0234 (10)	0.0205 (10)	0.0017 (8)	0.0054 (8)	0.0024 (8)
C2	0.0244 (10)	0.0221 (9)	0.0228 (10)	−0.0013 (8)	0.0069 (8)	0.0015 (8)
C3	0.0216 (10)	0.0170 (8)	0.0209 (9)	−0.0008 (7)	0.0053 (8)	−0.0034 (7)
C4	0.0214 (9)	0.0199 (9)	0.0182 (9)	−0.0037 (8)	0.0058 (7)	0.0013 (8)
C5	0.0300 (11)	0.0221 (10)	0.0222 (10)	0.0009 (8)	0.0059 (8)	−0.0024 (8)
C6	0.0371 (12)	0.0341 (12)	0.0196 (10)	−0.0042 (10)	−0.0004 (9)	−0.0029 (9)
C7	0.0294 (11)	0.0317 (11)	0.0265 (11)	−0.0032 (9)	−0.0011 (9)	0.0076 (9)
C8	0.0286 (11)	0.0244 (10)	0.0345 (12)	0.0022 (9)	0.0035 (9)	0.0022 (9)
C9	0.0273 (11)	0.0232 (10)	0.0278 (11)	0.0018 (8)	0.0028 (9)	−0.0044 (8)
C10	0.0289 (12)	0.0323 (12)	0.0465 (14)	0.0067 (10)	0.0119 (10)	0.0065 (11)
C11	0.0215 (10)	0.0238 (10)	0.0345 (11)	−0.0008 (8)	0.0089 (9)	0.0046 (9)
C12	0.0257 (10)	0.0272 (10)	0.0255 (10)	−0.0010 (9)	0.0068 (8)	−0.0009 (9)
C13	0.0229 (10)	0.0264 (10)	0.0214 (10)	−0.0005 (8)	0.0031 (8)	0.0022 (8)
C14	0.0210 (10)	0.0216 (9)	0.0228 (10)	−0.0027 (8)	0.0035 (8)	0.0011 (8)
C15	0.0242 (10)	0.0289 (11)	0.0221 (10)	−0.0010 (8)	0.0028 (8)	0.0019 (8)
C16	0.0229 (10)	0.0327 (11)	0.0289 (11)	0.0016 (9)	0.0013 (8)	0.0082 (9)

### 1-[(4-Methylphenyl)sulfonyl]-2-phenylazetidine Geometric parameters (Å, º)

**Table:**

S1—O1	1.4340 (14)	C7—H7	0.9500
S1—O2	1.4361 (14)	C7—C8	1.385 (3)
S1—N1	1.6319 (16)	C8—H8	0.9500
S1—C14	1.761 (2)	C8—C9	1.384 (3)
N1—C1	1.500 (2)	C9—H9	0.9500
N1—C3	1.499 (2)	C10—H10A	0.9800
C1—H1A	0.9900	C10—H10B	0.9800
C1—H1B	0.9900	C10—H10C	0.9800
C1—C2	1.539 (3)	C10—C11	1.509 (3)
C2—H2A	0.9900	C11—C12	1.390 (3)
C2—H2B	0.9900	C11—C16	1.394 (3)
C2—C3	1.552 (3)	C12—H12	0.9500
C3—H3	1.0000	C12—C13	1.385 (3)
C3—C4	1.496 (3)	C13—H13	0.9500
C4—C5	1.390 (3)	C13—C14	1.393 (3)
C4—C9	1.394 (3)	C14—C15	1.388 (3)
C5—H5	0.9500	C15—H15	0.9500
C5—C6	1.386 (3)	C15—C16	1.387 (3)
C6—H6	0.9500	C16—H16	0.9500
C6—C7	1.384 (3)		
			
O1—S1—O2	120.64 (9)	C7—C6—H6	119.9
O1—S1—N1	106.31 (8)	C6—C7—H7	120.1
O1—S1—C14	107.76 (9)	C6—C7—C8	119.7 (2)
O2—S1—N1	105.86 (8)	C8—C7—H7	120.1
O2—S1—C14	108.26 (9)	C7—C8—H8	120.0
N1—S1—C14	107.35 (9)	C9—C8—C7	119.9 (2)
C1—N1—S1	122.62 (13)	C9—C8—H8	120.0
C3—N1—S1	122.02 (12)	C4—C9—H9	119.5
C3—N1—C1	91.48 (14)	C8—C9—C4	120.92 (19)
N1—C1—H1A	114.0	C8—C9—H9	119.5
N1—C1—H1B	114.0	H10A—C10—H10B	109.5
N1—C1—C2	87.70 (14)	H10A—C10—H10C	109.5
H1A—C1—H1B	111.2	H10B—C10—H10C	109.5
C2—C1—H1A	114.0	C11—C10—H10A	109.5
C2—C1—H1B	114.0	C11—C10—H10B	109.5
C1—C2—H2A	114.0	C11—C10—H10C	109.5
C1—C2—H2B	114.0	C12—C11—C10	120.71 (19)
C1—C2—C3	88.04 (14)	C12—C11—C16	118.45 (19)
H2A—C2—H2B	111.2	C16—C11—C10	120.83 (19)
C3—C2—H2A	114.0	C11—C12—H12	119.3
C3—C2—H2B	114.0	C13—C12—C11	121.36 (19)
N1—C3—C2	87.29 (13)	C13—C12—H12	119.3
N1—C3—H3	110.3	C12—C13—H13	120.4
C2—C3—H3	110.3	C12—C13—C14	119.25 (18)
C4—C3—N1	116.72 (15)	C14—C13—H13	120.4
C4—C3—C2	119.91 (16)	C13—C14—S1	119.32 (15)
C4—C3—H3	110.3	C15—C14—S1	120.18 (15)
C5—C4—C3	119.08 (17)	C15—C14—C13	120.43 (19)
C5—C4—C9	118.51 (18)	C14—C15—H15	120.3
C9—C4—C3	122.40 (17)	C16—C15—C14	119.42 (19)
C4—C5—H5	119.7	C16—C15—H15	120.3
C6—C5—C4	120.64 (19)	C11—C16—H16	119.5
C6—C5—H5	119.7	C15—C16—C11	121.09 (19)
C5—C6—H6	119.9	C15—C16—H16	119.5
C7—C6—C5	120.26 (19)		
			
S1—N1—C1—C2	−148.20 (13)	C2—C3—C4—C9	77.7 (2)
S1—N1—C3—C2	148.51 (13)	C3—N1—C1—C2	−17.94 (14)
S1—N1—C3—C4	−89.15 (19)	C3—C4—C5—C6	178.66 (18)
S1—C14—C15—C16	176.57 (16)	C3—C4—C9—C8	−179.05 (19)
O1—S1—N1—C1	177.47 (14)	C4—C5—C6—C7	0.5 (3)
O1—S1—N1—C3	61.59 (16)	C5—C4—C9—C8	−0.1 (3)
O1—S1—C14—C13	−25.47 (18)	C5—C6—C7—C8	−0.1 (3)
O1—S1—C14—C15	157.66 (15)	C6—C7—C8—C9	−0.3 (3)
O2—S1—N1—C1	−53.09 (16)	C7—C8—C9—C4	0.4 (3)
O2—S1—N1—C3	−168.98 (14)	C9—C4—C5—C6	−0.3 (3)
O2—S1—C14—C13	−157.47 (15)	C10—C11—C12—C13	178.42 (19)
O2—S1—C14—C15	25.65 (19)	C10—C11—C16—C15	−178.3 (2)
N1—S1—C14—C13	88.67 (17)	C11—C12—C13—C14	0.0 (3)
N1—S1—C14—C15	−88.21 (17)	C12—C11—C16—C15	0.8 (3)
N1—C1—C2—C3	17.31 (14)	C12—C13—C14—S1	−176.44 (15)
N1—C3—C4—C5	155.55 (17)	C12—C13—C14—C15	0.4 (3)
N1—C3—C4—C9	−25.5 (3)	C13—C14—C15—C16	−0.3 (3)
C1—N1—C3—C2	17.79 (14)	C14—S1—N1—C1	62.37 (16)
C1—N1—C3—C4	140.14 (16)	C14—S1—N1—C3	−53.52 (16)
C1—C2—C3—N1	−17.33 (14)	C14—C15—C16—C11	−0.3 (3)
C1—C2—C3—C4	−136.81 (17)	C16—C11—C12—C13	−0.6 (3)
C2—C3—C4—C5	−101.2 (2)		
